# Bioactive properties of fucoidan from Malaysian brown seaweed (*Sargassum binderi*) with an assessment of its anti-diabetic potential in 3T3-L1 adipocytes

**DOI:** 10.1007/s13197-025-06410-z

**Published:** 2025-08-19

**Authors:** Nur Akmal Solehah Din, Piyapat Aiemcharoen, Decha Sermwittayawong, Seng Joe Lim, Ainaatul Asmaa’ Ishak, Noor-Soffalina Sofian-Seng, Noorul Syuhada Mohd Razali, Hafeedza Abdul Rahman, Wan Aida Wan Mustapha

**Affiliations:** 1 Department of Food Sciences, Faculty of Science and Technology, Universiti Kebangsaan Malaysia, Bangi, 43600 Selangor Malaysia; 2 Faculty of Fisheries and Food Science, Universiti Malaysia Terengganu, Kuala Nerus, Terengganu, 21030 Malaysia; 3 Division of Health and Applied Sciences, Faculty of Science, Prince of Songkla University, Hatyai, 90110 Songkhla Thailand; 4 Center of Excellence for Biochemistry, Faculty of Science, Prince of Songkla University, Hatyai, 90110 Songkhla Thailand; 5 Innovation Centre for Confectionery Technology (MANIS), Faculty of Science and Technology, Universiti Kebangsaan Malaysia, Bangi, 43600 Selangor Malaysia

**Keywords:** Fucoidan, Antioxidant activities, Anti-diabetic properties, Glucose uptake, Lipid accumulation

## Abstract

Fucoidan, a bioactive polysaccharide found in brown seaweed, is widely studied for its diverse health-promoting properties, offering promising potential for managing metabolic disorders, including oxidative stress and diabetes. This study investigates the physicochemical characteristics, antioxidant, and anti-diabetic properties of fucoidan extracted from *Sargassum binderi* (F_sar_). Fourier transform infrared spectroscopy (FTIR) spectrum confirmed the presence of key functional groups, with F_sar_ containing fucose and sulfate at 30.53% and 22.12%, respectively. In comparison, the commercial fucoidan (F_ysk_) exhibited significantly lower fucose (27.94%) but higher sulfate (26.28%) content. F_sar_ exhibited an equivalent total phenolic content (TPC; 34.32 mg GAE/g) and demonstrated comparable antioxidant activities, including 1,1-diphenyl-2-picrylhydrazyl (DPPH; IC_50_ 1.91 mg/mL), superoxide anion (SOA; 14.96%), and hydroxyl radical (•OH; 54.89%) scavenging activities, relative to F_ysk_. In anti-diabetic studies using 3T3-L1 adipocytes, F_sar_ showed a dose-dependent increase in glucose uptake, with a 65% enhancement observed at 0.5 mg/mL, surpassing the effect of standard insulin. Additionally, F_sar_ significantly inhibited lipid accumulation in adipocytes, with an approximate 66% reduction at the same concentration. These findings suggest that fucoidan from the underutilized Malaysian brown seaweed not only possesses strong antioxidant properties but also holds potential as a natural therapeutic agent for managing related metabolic disorders particularly, as an anti-diabetic.

**Supplementary Information:**

The online version contains supplementary material available at 10.1007/s13197-025-06410-z.

## Highlights

Fucoidan extracted from *S. binderi (F*_*sar*_*)* contains important functional groups and bioactive elements, making it a promising natural extract.

F_sar_ demonstrated strong antioxidant properties, aligning with the performance of commercial fucoidan (F_ysk_).

The extract significantly increased glucose uptake in cells, suggesting potential benefits for managing diabetes.

F_sar_ reduced lipid buildup in adipocytes, indicating its potential role in regulating fat metabolism.

**Supplementary Information:**

The online version contains supplementary material available at 10.1007/s13197-025-06410-z.

## Introduction

Fucoidan, a sulfated polysaccharide found in brown seaweeds is of particular interest due to its diverse biological activities. In Malaysia, the seaweed industry is substantial with an annual cultured production of approximately 225,048 metric tonnes valued at RM100 million in 2023 (NST Regional 2024), brown seaweed of *Sargassum binderi* remains underutilized due to its low market value and challenging sensory characteristics. In addition to being underexploited, the accumulation of this seaweed along coastlines can lead to environmental issues, such as the degradation of marine ecosystems and the production of hydrogen sulfide gas as it decomposes, which can harm local marine life and affect tourism (Van Tussenbroek et al. 2017). Converting this seaweed into valuable compounds (i.e. fucoidan) could mitigate these environmental impacts while also addressing economic challenges. This approach aligns with Malaysia’s sustainable development goals by transforming an environmental liability into a valuable resource that supports both ecological health and economic growth (Isa et al. 2022). Additionally, the development of seaweed-based industries plays a crucial role in generating employment opportunities, particularly in coastal communities. This integrated strategy aligns with circular economy initiatives in the ASEAN region by strengthening the biotechnology sector and addressing regional challenges related to sustainability and climate resilience (Herrador and Van 2024).

The phytochemical properties of fucoidan, including its fucose, sulfate, and total phenol content, play a critical role in its bioactivity. Fucose, a deoxyhexose sugar, is integral to the structure of fucoidan and contributes to its biological functions (Aziz et al. 2024; Khalafu et al. 2017). Sulfate groups enhance the polysaccharide’s ability to interact with various biomolecules, facilitating its antioxidative and anti-inflammatory activities. The sulfation pattern and degree of sulfation are crucial in determining the bioactivity of fucoidan. The sulfate groups in fucoidan are believed to interact with proteins and other biomolecules, enhancing their ability to scavenge free radicals and reduce oxidative stress (Pozharitskaya et al. 2020). The total phenol content in fucoidan is also important, as phenolic compounds are known for their strong antioxidant properties, further augmenting the polysaccharide’s ability to combat oxidative stress. During the extraction process, phenolic compounds are tightly bound to fucoidan, which may further potentiate the antioxidant activity by stabilizing these phenols within the polysaccharide matrix, ensuring their bioavailability and efficacy (Jayapala et al. 2022; Jayawardena et al. 2022).

The rising prevalence of type 2 diabetes in Malaysia, where approximately 18% of adults are affected, emphasizes the critical need for therapies that address the underlying causes (Institute for Public Health 2020). Oxidative stress plays a central role in diabetes development by promoting inflammation, impairing insulin signalling, and damaging insulin-producing β-cells (Caturano et al. 2023). Traditional treatments often fail to effectively target these oxidative processes, but fucoidan, with its potent antioxidant properties, offers a promising solution. By neutralizing reactive oxygen species (ROS) and reducing oxidative stress, fucoidan helps protect against inflammation, enhance insulin sensitivity, and regulate blood glucose levels; key factors in diabetes management (Li et al. 2024). Additionally, fucoidan’s antioxidant effects reduce lipid accumulation and inhibit the differentiation of preadipocytes into mature fat cells, thereby limiting excessive fat buildup (Sharma et al. 2022a; Sharma et al. 2022b). This is particularly important as excess fat is closely linked to insulin resistance, a major contributor to the onset of type 2 diabetes (Lee et al. 2021). Therefore, fucoidan’s dual role as an antioxidant and anti-diabetic agent makes it a valuable substance for addressing the underlying causes of type 2 diabetes and improving overall metabolic health.

While these properties of fucoidan extract have been explored, there is still limited research on the specific mechanisms through which this polysaccharide from Malaysian brown seaweed, *S. binderi* produce the effects. Therefore, the objectives of this study were to comprehensively evaluate the bioactive properties of fucoidan extracted from *S. binderi*, focusing on its chemical structure, physicochemical properties as well as its antioxidant activities. Then, the study also assessed the anti-diabetic potential of fucoidan by investigating its effects on glucose uptake stimulation and lipid accumulation inhibition in 3T3-L1 adipocytes. This research sought to contribute to the fields of nutritional biochemistry and therapeutic development, with the potential to support the development of fucoidan as a functional ingredient in the management of related metabolic disorders.

## Materials and methods

### Raw sample Preparation

Fresh *S. binderi* seaweed was harvested from a wild source at Selakan Island in Semporna, Sabah, Malaysia (coordinates: 4°34’38.600"N, 118°40’29.200"E) in June 2022. The seaweed was thoroughly rinsed with clean seawater to remove any dirt, sand, and other impurities. It was then sun-dried for 6 h before being transported to the laboratory and further used for fucoidan extraction. The seaweed sample was pre-washed thoroughly with distilled water, followed by a wash with 85% ethanol (HmbG Chemicals, Germany) to remove salts, contaminants, and impurities, and then dried at 40 °C in an oven for 72 h. The dried sample was cut into smaller pieces using a knife mill, then ground into a powder with a pulverizer and passed through a 0.5 mm sieve. The powdered sample was stored at 4 °C for later use. A commercial food-grade fucoidan (Yaizu Suisankagaku, Japan) with 92% purity (F_ysk_) was used as a standard in the present study.

### Extraction of fucoidan

Fucoidan extraction followed the method described by Fauziee et al. (2021), with slight modifications in terms of the number of cycles performed during the extraction procedure. Approximately 20 g of the sample (3 different sampling batches) was hydrolyzed in 250 mL of distilled water at 85 °C (200 rpm) for 2 h in a water bath shaker. The extract was filtered using a mesh (200–250 microns) to separate the liquid extract from the solid residue. Then, it was purified by removing alginate through treatment with calcium carbonate (CaCO_3_) at a ratio of 1 g CaCO_3_ to 10 mL crude extract in an incubator shaker at 50 °C and 200 rpm for 3 h, a process repeated three cycles. After filtration and centrifugation at 600 rpm for 15 min, the supernatant was ultra-filtrated with a 10 kDa molecular weight cut-off (MWCO) dialysis cassette to isolate fucoidan. The resulting F_sar_ extract was freeze-dried to remove any remaining solvent and stored in the freezer (– 40 ± 5 °C) until further use.

### Physicochemical and antioxidant properties

#### Chemical structure analysis

The structural analyses on fucoidans were conducted using Fourier Transform Infrared (FTIR) spectroscopy (PerkinElmer Spectrum 400 FT-IR/ FT-NIR Spectrometer, USA) fitted with a Spectrum Universal ATR Accessory. The spectra were recorded based on a scanning range of 4000–650 cm^− 1^ and a resolution of 4 cm^− 1^. The vibrational spectra were collected and analyzed using Essential FTIR software version 1.1.0.0.

#### Sulfate content assay

The sulfate content of fucoidans was conducted according to Khalafu et al. (2017). The fucoidan sample was prepared at 2 mg/mL in 1 M HCl (Merck, Germany). The sample was hydrolyzed at 105–110℃ for 5 h and cooled to room temperature. For each sample, two sets of mixtures were prepared (set A and set B), and both sets contained 0.2 mL of the sample hydrolysate and 3.8 mL of 3% (w/v) trichloroacetic acid (Merck, Germany). About 1.0 mL of barium chloride-gelatine solution (0.5% w/v barium chloride (Merck, Germany) in 0.5% w/v gelatine (Sigma-Aldrich, Germany) solution) and 1.0 mL of gelatine (Sigma-Aldrich, Germany) solution (0.5% w/v) were added to set A and set B, respectively. The solution of both sets was mixed for 20 min at room temperature. Next, 200 µL of the sample was transferred to a microplate, and the absorbance was measured at 396 nm using a microplate spectrophotometer (Biotek Epoch, USA) against the corresponding reagent blanks, set A and set B without the sample. Serial dilutions of potassium sulfate (K_2_SO_4_; 0–100 µg/mL) standard (Sigma-Aldrich, Germany) were also prepared and measured against set A reagent blank. Lastly, the sulfate content of the sample was calculated based on the K_2_SO_4_ calibration curve and the analysis was performed in triplicate (*n* = 3).

#### Fucose content assay

The fucose analysis was conducted according to Khalafu et al. (2017). Approximately 200 µL of the fucoidan sample (2 mg/mL) was pipetted into the microcentrifuge tube, followed by the addition of 900 µL of diluted sulphuric acid (Merck, Germany) (6 H_2_SO_4_: 1 H_2_O). The mixture was placed in a boiling water bath (100℃) for 10 min and transferred to an ice-water bath for 5 min. It was allowed to cool to room temperature, and 50 µL of 3% (w/v) L-cysteine (Sigma-Aldrich, Germany) HCl solution was subsequently added. The solution was mixed and allowed to stand for 30 min before being transferred (200 µL) to a microplate. The absorbance was measured at 396 nm and 427 nm using a microplate spectrophotometer. The sample without L-cysteine HCl was used as the blank. The difference in absorbance between the two wavelengths (λ_396_– λ_427_) was used to differentiate fucose from other hexoses in the sample (Lim et al. 2014). A standard curve of L-fucose (0.01–0.1 mg/mL (Merck, Germany) was used to calculate the fucose content in the fucoidan sample. The fucose analysis was conducted in triplicate (*n* = 3).

#### Total phenolic content (TPC) assay

The analysis of TPC in the fucoidan sample was determined based on the Folin-Ciocalteu method described by Isa et al. (2022). About 0.3 mL of sample (2 mg/mL) was mixed with 1.5 mL of the Folin-Ciocalteu reagent (Supelco, Germnay) and 1.2 mL of 7.5% (w/v) sodium carbonate (Na_2_CO_3_) (Merck, Germany) solution in a test tube. The reaction mixture was incubated for 90 min at room temperature in the dark. Approximately 200 µL of the mixture was transferred to a microplate, and the absorbance was measured at 765 nm using a microplate spectrophotometer. The gallic acid (Sigma-Aldrich, Germany) standard curve, with the concentration of 0–0.5 mg/mL was constructed to calculate the TPC of the fucoidan sample; expressed as mg gallic acid equivalent per g sample (mg GAE/ g). The TPC analysis was conducted in triplicate (*n* = 3).

#### Free radical 1,1-diphenyl-2- Picrylhydrazyl (DPPH) scavenging assay

The fucoidan sample’s scavenging ability of stable 1,1-diphenyl-2- picrylhydrazyl (DPPH) radical was conducted according to Lim et al. (2014). The fucoidan sample was prepared at a specific range (0–2.5 mg/mL), and 1.0 mL of each fucoidan concentration was mixed with 2.9 mL of 0.15 mM methanolic DPPH (Sigma-Aldrich, Germany) in a test tube. The mixture was incubated for 30 min at room temperature in the dark. Approximately 200 µL of the mixture was transferred to a microplate, and the absorbance was measured at 517 nm using a microplate spectrophotometer. The DPPH inhibition (%) was calculated based on Eq. 1:

1 $$\begin{gathered}{\mathrm{DPPH}}\,{\mathrm{free}}\,{\mathrm{radical}}\,{\mathrm{scavenging}}\,{\mathrm{activity}}\,\left( \% \right)\, \\ {\text{ = }}\,\left[ {\left( {{{\mathrm{A}}_{\mathrm{1}}}{\text{ - }}\,{{\mathrm{A}}_{\mathrm{2}}}} \right){\mathrm{/}}{{\mathrm{A}}_{\mathrm{1}}}} \right] \times 100 \\ \end{gathered} $$

Where A_1_ is the absorbance of the DPPH blank (without the sample) and A_2_ is the absorbance of the sample with DPPH. The IC_50_ of the fucoidan sample was determined based on a plot of fucoidan concentration (mg/mL) versus DPPH inhibition (%), where the IC_50_ value was estimated from the fitted line. The DPPH assay was performed in triplicate (*n* = 3).

#### Superoxide anion (SOA) scavenging assay

The analysis methodology was conducted according to Fauziee et al. (2021). The inhibition of pyrogallol autoxidation was measured to determine fucoidan’s superoxide anion scavenging activity. About 0.3 mL of sample (2 mg/mL) was mixed with 2.6 mL of 50 mM phosphate buffer (pH 8.24) and 90 mL of 3 mM pyrogallol (Sigma-Aldrich, Germany) solution, and the mixture was dissolved in 10 mM HCl (Merck, Germany) in the test tube. The mixture (200 µL) was transferred to a microplate, and the absorbance was measured at 517 nm using a microplate spectrophotometer. The inhibition rate of pyrogallol autoxidation was reflected by the absorbance recorded every minute (time interval) throughout the 10 min reaction time. The SOA scavenging activity was calculated based on Eq. 2:

2 $${\mathrm{SOA}}{\mkern 1mu} {\mathrm{scavenging}}{\mkern 1mu} {\mathrm{rate}}{\mkern 1mu} \left( \% \right){\mkern 1mu} \,{\text{ = }}\,{\mkern 1mu} \left[ {{\mathrm{1}}\,{\text{ - }}\,\left( {{{\mathrm{A}}_{\mathrm{2}}}\,{\text{ - }}\,{{\mathrm{A}}_{\mathrm{1}}}} \right){\mathrm{/}}{{\mathrm{A}}_{\mathrm{0}}}} \right]\, \times \,{\mathrm{100}}$$

Where A_0_ is the autoxidation rate of pyrogallol and the absorbance changes in the blank from 0 to 10 min, A_1_ is the sample absorbance at 0 min, and A_2_ is the sample absorbance at 10th min. An *n* = 3 was conducted on the SOA scavenging activity.

#### Hydroxyl radical (•OH) scavenging assay

The analysis was performed according to the method reported by Fauziee et al. (2021) and Khalafu et al. (2017). Firstly, 0.5 mL of 9 mM ferrous sulfate (FeSO_4_) (Sigma-Aldrich, Germany) solution was mixed with 1.0 mL of 8.8 mM hydrogen peroxide (H_2_O_2_) (Merck, Germany) solution. The reaction mixture can generate OH radicals through the Fenton reaction mechanism. Then, two sets of tests were performed (set A and set B). For set A, 50 µL of the sample (2 mg/mL) was added to the reaction mixture, followed by 0.2 mL of 9 mM salicylic acid (Sigma-Aldrich, Germany) solution. Meanwhile, for set B, only 50 µL of the sample (2 mg/mL) was added to the mixture. Both test sets were allowed to stand at 37℃ for 1 h. The test mixture (200 µL) was transferred to a microplate, and the absorbance was measured at 510 nm using a microplate spectrophotometer. The •OH scavenging activity was calculated based on Eq. 3:

3 $$ \cdot {\mathrm{OH}}\,{\mathrm{scavenging}}\,{\mathrm{rate}}\,\left( \% \right){\mkern 1mu} \,{\text{ = }}\,{\mkern 1mu} \left[ {{\mathrm{1}}\, - \,\left\{ {\left( {{{\mathrm{A}}_{\mathrm{1}}}{\mathrm{0}}{{\mathrm{A}}_{\mathrm{2}}}} \right){\mathrm{/}}{{\mathrm{A}}_{\mathrm{0}}}} \right\}} \right]\, \times \,{\mathrm{100}}$$

Where A_0_ is the absorbance of the blank, i.e., the reaction mixture without sample, A_1_ is the absorbance of the set A test, and A_2_ is the absorbance of the set B test. The analysis was performed in triplicate (*n* = 3).

### Culturing of 3T3-L1 adipocytes

The mouse 3T3-L1 preadipocyte cells (ATCC CL-173™) were cultured and induced to differentiate into adipocytes as previously described (Nuinamwong et al. 2024; Sim et al. 2019). Cells were cultured in low-glucose Dulbecco’s Modified Eagle Medium (DMEM) (Invitrogen, USA) supplemented with 10% fetal bovine serum (FBS) (Gibco, Australia) and antibiotics (Gibco, USA), with subculturing performed every 2 days. Cells were plated and allowed to reach 80–90% confluence. To induce differentiation, 2 days after reaching confluence (Day 0), 3T3-L1 pre-adipocytes were treated with differentiation activation (D/A) medium, which consisted of high-glucose DMEM (Invitrogen, USA) with 10 µg/mL insulin (Sigma-Aldrich, Germany), 0.25 µM dexamethasone (Sigma-Aldrich, Germany), 500 µM 1-methyl-3-isobutylxanthine (IBMX) (Sigma-Aldrich, Germany), 10% FBS (Gibco, Australia), and penicillin-streptomycin (Gibco, USA). Three days later (Day 4), the medium was replaced with a differentiation maintain (D/M) medium, containing high-glucose DMEM (Invitrogen, USA), 10 µg/mL insulin (Sigma-Aldrich, Germany), 10% FBS (Gibco, Australia), and penicillin-streptomycin (Gibco, USA). The D/M medium was refreshed for 2 days. By the end of the differentiation period, cells developed into mature adipocytes with observable lipid droplets under a light microscope. All cultures were maintained at 37 °C in a 5% CO_2_ incubator (Binder C170, Germany).

### Anti-diabetic properties

#### Cell viability assay

The viability of fucoidan-treated cells was assessed using 3-(4,5-dimethylthiazol-2-yl)-2,5-diphenyl tetrazolium bromide (MTT) reduction assay, as previously described by Nuinamwong et al. (2024). After treatment with fucoidan at different concentrations (0.005, 0.05, 0.5 mg/mL) for 24 h at the desired concentration, differentiated cells were washed once with 1× PBS (Sigma-Aldrich, Germany). Subsequently, the cells were incubated with 200 µL of MTT (Invitrogen, USA) solution (0.5 mg/mL) for 4 h at 37 °C in a 5% CO_2_ incubator. Following incubation, the supernatant was carefully aspirated, and 200 µL of dimethyl sulfoxide (DMSO) (Sigma-Aldrich, Germany) was added to solubilize the formazan crystals. Absorbance was measured using a microplate spectrophotometer at 570 nm, and cell viability was expressed as a percentage of the control (*n* = 3). The antidiabetic drugs of insulin (Gibco, USA) and metformin (Sigma-Aldrich, Germany) were used as positive controls.

#### Glucose uptake assay

To directly measure the glucose uptake into the cells, a glucose analogue of 2-deoxy-2-[(7-nitro-2,1,3-benzoxadiazol-4-yl) amino]-D-glucose (2-NBDG) was used according to Nuinamwong et al. (2024). The differentiated cells were subjected to serum starvation for 18 h using serum-free DMEM (Invitrogen, USA) with 0.5% bovine serum albumin (BSA) (Gibco, USA). After this period, the cells were rinsed once with 1× PBS (Sigma-Aldrich, Germany) and then treated with fucoidan. The cells were collected after 24 h of treatment. Subsequently, 200 µM of 2-NBDG (Invitrogen, USA) was added to the cells, and incubation continued for 10 min. Then, the cells were washed three times with ice-cold 1× PBS (Sigma-Aldrich, Germany) and then lysed with 150 µL of 1% Triton X-100 (Thermo Fisher Scientific, USA). The fluorescence signal of 2-NBDG was measured using a fluorescence microplate reader with an excitation wavelength of 465 nm and an emission wavelength of 540 nm, and glucose uptake was expressed as a percentage of the control (*n* = 3). The antidiabetic drugs of insulin (Gibco, USA) and metformin (Sigma-Aldrich, Germany) were used as positive controls.

#### Lipid accumulation assay

The lipid accumulation assay was carried out by introducing fucoidan at the early stage of 3T3-L1 adipogenic differentiation, following the procedure outlined by Sim et al. (2019). Fucoidan was added with the D/A medium at the onset of differentiation and with the D/M medium throughout the differentiation process. On the 12th day of differentiation, cells were washed twice with 1× PBS (Sigma-Aldrich, Germany) and then fixed with 4% paraformaldehyde (Thermo Fisher Scientific, USA). After washing with 60% isopropanol (Thermo Fisher Scientific, USA), the cells were air-dried and stained with 0.3% Oil-Red O dye (Sigma-Aldrich, USA) (dissolved in 60% isopropanol) for 10 min. Following staining, cells were washed once with water and air-dried, after which photographs of the stained cells on the plate were taken. To quantify the Oil-Red O dye, DMSO (Sigma-Aldrich, Germany) was added to the cells, and the absorbance at 540 nm was measured (*n* = 3). Tumour necrosis factor-alpha (TNF-α) (Gibco PeproTech, USA) was used as a control to inhibit lipid accumulation, while rosiglitazone (Sigma-Aldrich, Germany) was used as a control to promote lipid accumulation. Whereas undifferentiated cells served as a control for non-differentiated cells.

### Statistical data analysis

Experimental data obtained were expressed as mean ± standard deviation. The data were analyzed using one-way analysis of variance (ANOVA) with Tukey’s post hoc test and a significant difference of *p* < 0.05 using Minitab statistical software version 17.

## Results and discussion

### Chemical characterization and bioactive properties

**Fig. 1:**
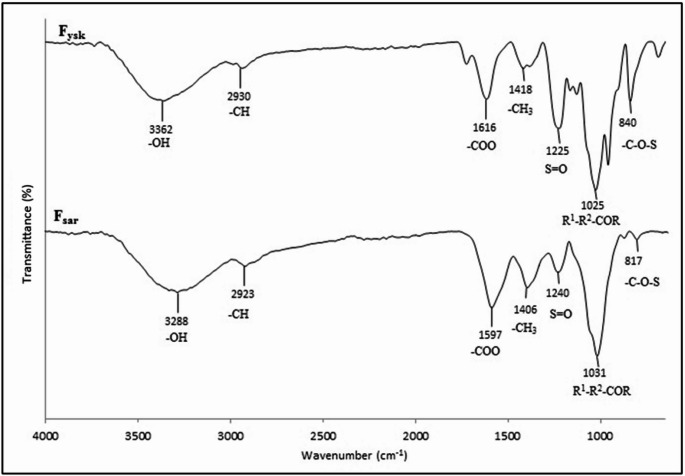
FTIR spectrum of commercial fucoidan (F_ysk_) and extracted fucoidan (F_sar_)

The chemical composition of fucoidan from Malaysian *S. binderi* (F_sar_) was confirmed by comparing the functional groups present in the extract to that of commercial fucoidan (F_ysk_), as illustrated in the FTIR spectrum (Fig. 1). In the present study, F_ysk_ (92% purity) was used as a standard to assess the functional qualities of the extracted F_sar_ and determine its relative quality as a high-value substance. Our previous study reported that the purity of the fucoidan extract in the present study was 78.30% (Din et al. 2024). The broad absorption bands around 3288 cm^-1^ for F_sar_ and 3362 cm^-1^ for F_ysk_ correspond to hydroxyl (-OH) groups, typical of polysaccharides. The slight shift in wavenumber suggests differences in the hydrogen bonding environment between the two samples. Aliphatic C-H stretching vibrations are observed at 2923 cm^-1^ for F_sar_ and 2930 cm^-1^ for F_ysk_, indicating the presence of -CH_2_- groups (Hong et al. 2021). The peaks associated with carboxylate anions (-COO-) at 1597 cm^-1^ and 1406 cm^-1^ in F_sar_, and at 1616 cm^-1^ and 1418 cm^-1^ in F_ysk_, point to the presence of uronic acid residues. Fucoidans derived from several species of brown seaweed, such as *Fucus vesiculosus* have simple chemical compositions, primarily consisting of fucose and sulfate. However, the chemical compositions of most fucoidans are more complex and often include other monosaccharides (i.e. mannose, galactose, glucose, and xylose), as well as uronic acids, acetyl groups, and even proteins (Jayawardena et al. 2019).

Sulfate groups, a crucial component of fucoidans, are confirmed by the significant peaks at 1240 cm^-1^ for F_sar_ and 1225 cm^-1^ for F_ysk_, attributed to S = O stretching vibrations (Khalafu et al. 2017). These differences may suggest variations in the degree of sulfation or the surrounding molecular environment (Jayapala et al. 2022). Both samples show consistent peaks at 1025 cm^-1^, indicative of glycosidic linkages (C-O-C), indicating a similar polysaccharide backbone structure; associated with the fucose content of fucoidan (Fauziee et al. 2021). Additional peaks at 817 cm^-1^ and 1031 cm^-1^ in F_sar_, and corresponding peaks at 840 cm^-1^ and 1031 cm^-1^ in F_ysk_, further confirm the presence of sulfate ester groups or different substitution patterns within the polysaccharide structure (Tan et al. 2020). As FTIR spectra confirmed the presence of important chemical components in both fucoidan samples, further substantiation regarding the content of fucose, sulfate, and the antioxidant properties of fucoidans were examined. Table 1 shows the physicochemical and antioxidant properties of F_ysk_ and F_sar_.

**Table 1:** Physicochemical and antioxidant properties of commercial fucoidan (F_ysk_) and extracted fucoidan (F_sar_)

Parameter	F_ysk_	F_sar_
Fucose (%)	27.94 ± 0.84^b^	30.53 ± 0.49^a^
Sulfate (%)	26.28 ± 1.01^a^	22.12 ± 0.71^b^
TPC (mg GAE/g)	34.45 ± 2.64^a^	34.32 ± 3.03^a^
DPPH (IC_50_; mg/mL)	1.74 ± 0.02^a^	1.91 ± 0.17^a^
SOA (%)	15.23 ± 1.31^a^	14.96 ± 1.58^a^
•OH (%)	56.00 ± 3.64^a^	54.89 ± 4.02^a^

Values show mean ± standard deviation (*n* = 3) with different superscripts ^a–b^ is significantly different (*p* < 0.05)

The analysis of the physicochemical and antioxidant properties of both fucoidans in Table 1 reveals some notable differences. The fucose content of F_sar_ of 30.53% was observed to be significantly higher (*p* < 0.05) than the F_ysk_ (27.94%). A lower fucose content of 21.08% from *S. hystrix* was reported by Husni et al. (2022) using 2% CaCl_2_ (85℃, 4 h). Meanwhile, the fucoidan extracted using microwave-assisted extraction from *S. siliquosum* achieved the highest sulfate content of 32.1%, which is almost equivalent to that of the present study (Chen et al. 2021). Whereas the sulfate content of F_sar_ (22.12%) was observed significantly lower (*p* < 0.05) than F_ysk_ at 26.28%. Previously, Husni et al. (2022) reported that fucoidan extracted with 0.5% EDTA at 70 °C for 3 h had a sulfate content of 27.80%, comparable to the present study. In contrast, extraction with 2% CaCl_2_ at 85 °C for 4 h resulted in a lower sulfate content of 15.31%. Despite these differences in fucose and sulfate content, the total phenolic content (TPC) was insignificant different (*p* < 0.05) between the two samples, with F_sar_ at 34.32 mg GAE/g and F_ysk_ at 34.45 mg GAE/g. A study by Hanjabam et al. (2019) found that the TPC of crude fucoidan extract from *Sargassum wightii* could be in the range of 20.21–32.18 mg GAE/g.

Subsequently, the IC_50_ results of the DPPH assay were insignificant (*p* > 0.05) for both F_sar_ and F_ysk_, with 1.91 and 1.74 mg/mL, respectively. According to Fauziee et al. (2021), the hydrogen atom or electron transfer reaction from the active negatively-charged sulfate group of fucoidan to the DPPH free radical was the main reason for fucoidan DPPH scavenging activity. Meanwhile, fucose residues enhance antioxidant mechanisms in a different manner, promoting a flexible and branched molecular structure, increasing aqueous solubility, and aiding in the outward exposure of functional groups such as sulfate and hydroxyl groups of fucoidan (Wang et al. 2020). The DPPH IC_50_ of fucoidans in the present study were comparable to those reported by Ardiana and Husni (2020) with IC_50_ of 1.82 mg/mL, which was exhibited by fucoidan extracted from *Sargassum hystrix* using EDTA at 90℃. While the distinct variations in fucose and sulfate contents might influence their overall biological activities, the insignificant difference in their DPPH scavenging activity suggests that the primary antioxidant activity of F_sar_ is quite comparable to that of the commercial F_ysk_.

Analyses of its secondary antioxidant activities showed that the SOA scavenging activity and •OH scavenging activity of F_sar_ (SOA: 14.96% and •OH: 54.89%) are insignificantly different (*p* > 0.05) than F_ysk_ (SOA: 15.23% and •OH: 56.00%). A relatively comparable result by Fauziee et al. (2021) was reported on SOA (8.9–15.4%) and •OH (53.4–56.5%) scavenging activities of fucoidan from three different seaweeds of *Sargassum polycystum*, *Turbinaria ornate*, and *Padina boryana*. The sulfate groups in fucoidan help neutralize these radicals by donating electrons, converting them into less harmful substances like hydrogen peroxide (H_2_O_2_) or water. Besides, sulfate groups can prevent radical formation by chelating metal ions, thus inhibiting metal-catalysed oxidative reactions and reducing the generation of harmful free radicals (Khalafu et al. 2017; Lim et al. 2014). Then, fucose in fucoidan enhances the molecule’s flexibility and solubility, improving its ability to interact with free radicals and boosting antioxidant activity. Together, the electron donation from sulfate groups and the structural benefits provided by fucose make fucoidan an effective antioxidant (Wang et al. 2020).

Therefore, fucoidan can help protect cells from oxidative damage, contributing to its overall bioactivity and potential therapeutic benefits in managing oxidative stress-related conditions. As F_sar_ exhibited significant antioxidant activities that are equivalent to F_ysk_, it can promote the reduction in oxidative stress, which is highly related to the subsequent bioactive potentials. Hence, the fucoidan from *S. binderi* (F_sar_) was further used for an anti-diabetic study in 3T3-L1 adipocytes.

### Anti-diabetic properties in 3T3-L1 adipocytes

#### Cell viability and stimulation of glucose uptake

**Fig. 2:**
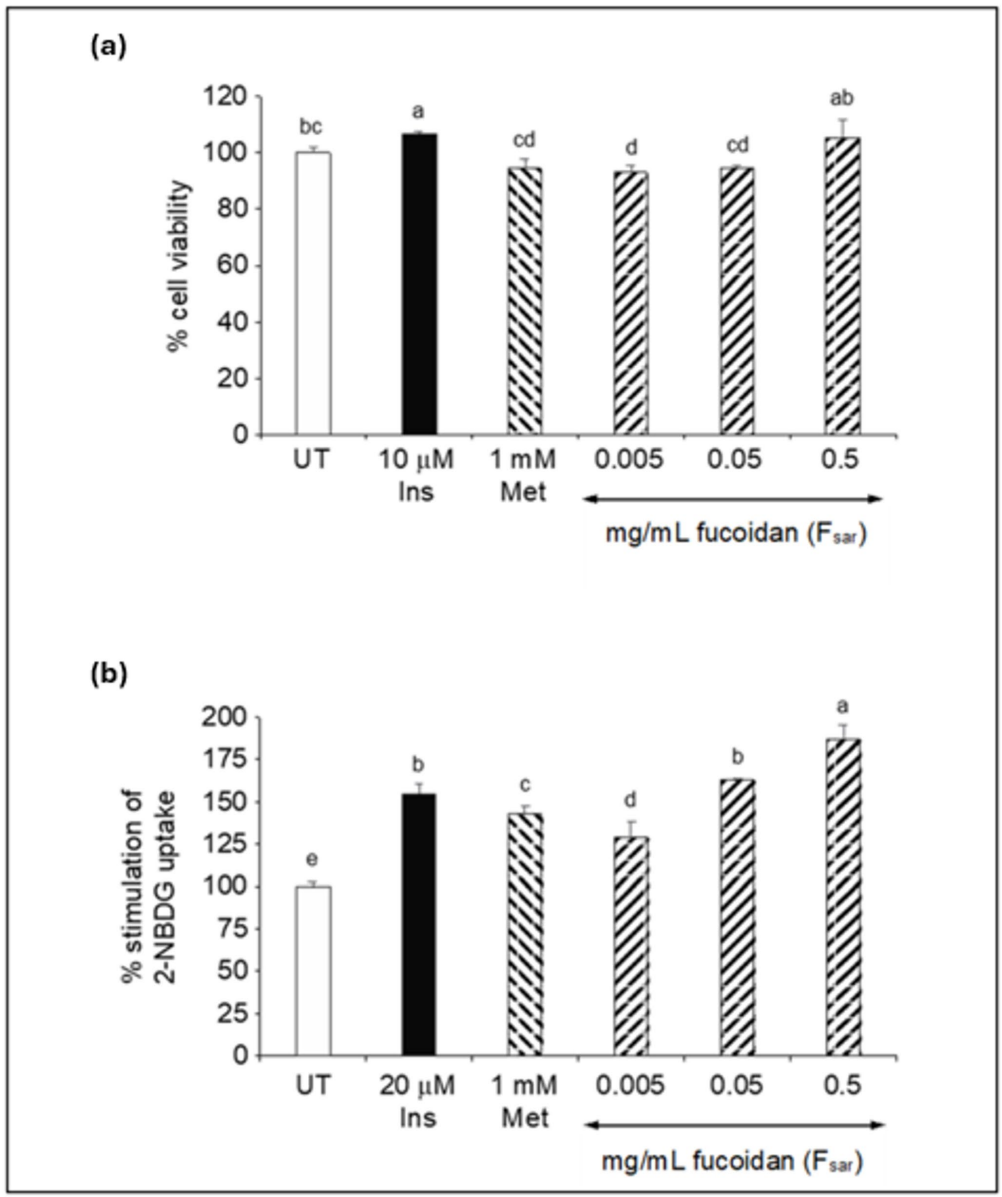
Fucoidan dose-dependently stimulated 2-NBDG uptake in 3T3-L1 adipocytes without affecting cellular viability. (**a**) shows cell viability in 3T-L1 cells incubated with F_sar_ at different concentrations (0.005–0.5 mg/mL) for 24 h. (**b**) shows 2-NBDG uptake in untreated cells, cells treated with 20 µM insulin, cells treated with 1 mM metformin, and cells treated with 0.005–0.5 mg/mL F_sar_ for 24 h. UT, Ins, and Met are abbreviated for untreated, insulin, and metformin, respectively. Both insulin and metformin as positive controls for stimulation of 2-NBDG uptake. Bars show mean ± standard deviation (*n* = 3) with different superscripts ^a–e^ is significantly different (*p* < 0.05)

Based on Fig. 2a, the results demonstrate the effects of F_sar_ on the viability of 3T3-L1 adipocytes, in comparison with standard anti-diabetic treatments (i.e. insulin and metformin). F_sar_ treatment at low concentrations (0.005–0.05 mg/mL) resulted in a notable decrease in cell viability (~ 93–95%) but, with an insignificant effect (*p* < 0.05) compared to the standard metformin (94.24%). A study by Park et al. (2011) observed a slight reduction in cell viability of 3T3-L1 cells when treated with fucoidan at 0.05–0.2 mg/mL compared to untreated though, the effect was reported to be insignificant (*p* > 0.05). Remarkably, at the highest concentration tested (0.5 mg/mL), F_sar_ did not reduce cell viability. Instead, its viability (105.44%) was restored to levels comparable to the untreated control and insulin-treated groups. This biphasic response suggests that at lower concentrations, fucoidan may induce slight stress in adipocytes, whereas, at higher concentrations, it could provide a supportive or no effect on cell survival (Shiau et al. 2022). This phenomenon may be related to the complexity of fucoidan’s molecular structure and its interaction with cellular pathways, which may differ at various concentrations (Wang et al. 2019). Thus, the observed effects of F_sar_ highlight the potential for dose-dependent modulation of cellular processes without affecting cellular viability, which could have implications for its use as a therapeutic agent in metabolic disorders, including diabetes.

The ability of F_sar_ to stimulate glucose uptake in 3T3-L1 adipocytes was further investigated using fluorescently labelled glucose, 2-NBDG (Fig. 2b). This result provides valuable insights into the potential of fucoidan to enhance glucose uptake, a critical factor in the management of diabetes. At a low concentration of 0.005 mg/mL, F_sar_ induces an approximately 10% increase in 2-NBDG uptake, which represents a significant enhancement compared to the control. This suggests that even at low concentrations, F_sar_ has a positive effect on glucose uptake. Similarly, at a high concentration of 0.5 mg/mL, F_sar_ induces a substantial increase in 2-NBDG uptake, reaching approximately 65%, which surpasses the effect of standard insulin (50%). A significant increase in 2-NBDG uptake of approximately 30% at 0.01 mg/mL fucoidan was reported by Sim et al. (2019), and the uptake showed an increasing trend with increasing fucoidan concentration; around 40% glucose uptake with 0.2 mg/mL fucoidan. This significant increase suggests that the fucoidan may activate additional pathways or enhance existing ones involved in glucose uptake.

Jeong et al. (2013) reported that fucoidan could stimulate 2-deoxyglucose (2-DG) uptake in L6 myotubes and promoted the phosphorylation of AMP-activated protein kinase (AMPK) in skeletal muscles of leptin receptor-deficient (db/db) mice and in L6 myotubes. These results suggest that AMPK activation may be a mechanism of fucoidan to promote glucose uptake in 3T3-L1 adipocytes. Stimulation of glucose uptake in 3T3-L1 adipocytes is not the only antidiabetic mechanism of fucoidan. Previous studies showed that fucoidan from several brown seaweeds such as *Sargassum wightii*, *Ecklonia radiata*, and *Tubinaria ornata* inhibited α-amylase enzyme (Mabate et al. 2021). Interestingly, a previous study showed that fucoidans from *Ascophyllum nodosum* and *Sargassum longicruris* with similar sulfate content (19–20%) to the present study were able to inhibit α-amylase (Kim et al. 2015). However, when the fucoidan from *Ascophyllum nodosum* was desulfated, the α-amylase inhibitory was abolished. These results suggest that a high sulfate content in fucoidan is required for the inhibition of α-amylase. Thus, fucoidan’s ability to stimulate glucose uptake at this level could imply its potential to enhance insulin sensitivity or act independently to improve glucose metabolism, indicating the potential antidiabetic activity of the polysaccharide.

#### Inhibition of lipid accumulation

**Fig. 3:**
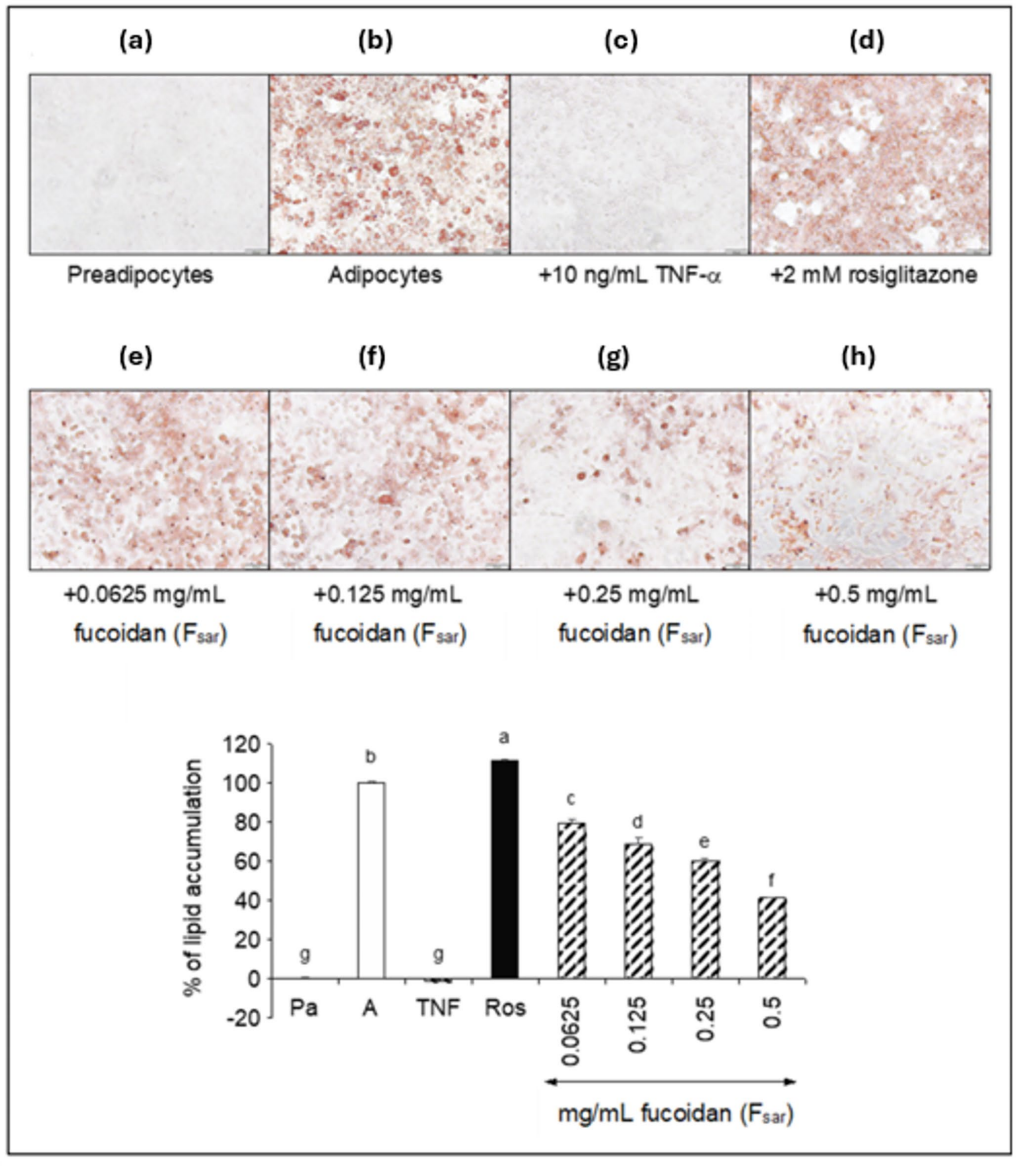
Fucoidan dose-dependently inhibited lipid accumulation in differentiating 3T3-L1 adipocytes. Panels (**a**)-(**h**) show the Oil-Red O-stained adipocytes that were treated from the start of differentiation with TNF-α (**c**), rosiglitazone (**d**), and an increasing dose of F_sar_ (**e**)-(**f**). Undifferentiated preadipocytes (**a**) and fully differentiated adipocytes (**b**) were included as the controls for the staining assay. The bar graph shows lipid accumulation calculated, and each bar indicated mean ± standard deviation (*n* = 3) with different superscripts ^a–f^ is significantly different (*p* < 0.05)

Results in Fig. 3 show that the cells treated with F_sar_ at 0.0625–0.5 mg/mL exhibited a decrease in lipid droplets in a dose-dependent manner. At the low F_sar_ concentrations (0.0625–0.125 mg/mL), there is a slight reduction in lipid content (34–40%), as evidenced by less intense staining and a significantly lower percentage of lipid accumulation (*p* < 0.05) compared to the control. The most substantial reduction in lipid accumulation is observed at the highest F_sar_ concentration (0.5 mg/mL). The staining is considerably less intense, and the data show a significant decrease (*p* < 0.05) in the lipid content with ~ 66% reduction, indicating that F_sar_ at this concentration strongly inhibits lipid accumulation in 3T3-L1 adipocytes. This aligns with previous findings on fucoidan’s anti-diabetic effects, where it has been shown to reduce body weight gain and fat mass in animal models (Wen et al. 2021). The study by Sim et al. (2019) reported the highest lipid accumulation reduction was approximately 55% using 0.2 mg/mL fucoidan in 3T3-L1 adipocytes. The overall dose-dependent reduction in lipid accumulation with F_sar_ treatment is particularly noteworthy. It suggests that fucoidan could be a potent natural compound for managing diabetes by inhibiting adipogenesis and reducing lipid storage in adipocytes.

Previous study suggests that fucoidan may interfere with key pathways involved in lipid storage, such as the peroxisome proliferator-activated receptor gamma (PPARγ) pathway, which is crucial for adipocyte differentiation and lipid metabolism (Xu et al. 2014). Fucoidan from *Fucus Vesiculosus* has been reported could suppress adipogenesis through the downregulation of CCAAR/enhancer-binding protein *α* (C/EBPα), proliferator-activated receptor *γ* (PPARγ), and adipocyte protein 2 (aP2) of both gene and protein expressions. Furthermore, the fucoidan also inhibited the activation of MAPK proteins, including p38 mitogen-activated protein kinases (p38MAPK), extracellular signal-regulated kinase (ERK), and c-Jun N-terminal kinases (JNKs) proteins (Kim et al. 2010). Similarly, fucoidan isolated from *Undaria pinnatifida* inhibited adipogenesis through the downregulation of PPARγ, C/EBPα, and aP2 genes and proteins (Sim et al. 2019). These findings highlight fucoidan’s promise as a natural therapeutic agent for combating diabetes and related metabolic disorders, justifying further investigation into its mechanisms of action and potential applications in clinical settings.

## Conclusion

The fucoidan extracted from *S. binderi* (F_sar_) exhibits potent antioxidants comparable to commercial fucoidan (F_ysk_). F_sar_’s higher fucose content and effective radical scavenging activities suggest its potential as a bioactive compound. In particular, F_sar_ demonstrated significant glucose uptake stimulation and lipid accumulation inhibition in 3T3-L1 adipocytes, indicating its capability to enhance insulin sensitivity and mitigate adipogenesis. These results support the use of F_sar_ as a natural therapeutic agent for the management of diabetes and related metabolic disorders, warranting further exploration into its mechanisms and potential clinical applications.

## Supplementary Information

Below is the link to the electronic supplementary material.

Supplementary Material 1

## Data Availability

All data and findings are provided in the article.

## Abbreviations

F_sar_: Fucoidan extracted from S. binderi
F_ysk_: Commercial food-grade from Yaizu Suisankagaku Industry
FTIR: Fourier transform infrared spectroscopy
TPC: Total phenolic content
GAE: Gallic acid equivalent
DPPH: 1,1-diphenyl-2-picrylhydrazyl
SOA: Superoxide anion
•OH: Hydroxyl radical
ROS: Reactive oxygen species
MWCO: Molecular weight cut-off
FBS: Fetal bovine serum
DMEM: Dulbecco’s Modified Eagle Medium
D/A: Differentiation activation
D/M: Differentiation maintain
IBMX: 1-methyl-3-isobutylxanthine
MTT: 3-(4,5-dimethylthiazol-2-yl)-2,5-diphenyl tetrazolium bromide
DMSO: Dimethyl sulfoxide
2-NBDG: 2-deoxy-2-[(7-nitro-2,1,3-benzoxadiazol-4-yl) amino]-D-glucose
2-DG: 2-deoxyglucose
TNF-α: Tumour necrosis factor-alpha
AMPK: AMP-activated protein kinase
C/EBPα: CCAAR/enhancer-binding proteinα
PPARγ: Proliferator-activated receptorγ
aP2: Adipocyte protein 2
p38MAPK: p38 mitogen-activated protein kinases
ERK: Extracellular signal-regulated kinase
JNKs: c-Jun N-terminal kinases
