# Supplementary material for: Bioactive properties of fucoidan from Malaysian brown seaweed (Sargassum binderi) with an assessment of its anti-diabetic potential in 3T3-L1 adipocytes
Source: J Food Sci Technol. 2025 Aug 19;63(1):156–67. doi: 10.1007/s13197-025-06410-z (PMC12891271; doi:10.1007/s13197-025-06410-z)
Supplement: Supplementary file 1 — Supplementary Material 1 [file 13197_2025_6410_MOESM1_ESM.pdf]

Prof. Ts. Dr. Wan Aida Wan Mustapha
National University of Malaysia (UKM)
43600 Selangor
Malaysia
wanaidawm@ukm.edu.my

Dr. N. Bhaskar
The Editor-in-Chief
Journal of Food Science and Technology

6th May 2025

Dear Editor,

REVISED MANUSCRIPT R1: BIOACTIVE PROPERTIES OF FUCOIDAN FROM MALAYSIAN BROWN SEAWEED (*SARGASSUM BINDERI*) WITH AN ASSESSMENT OF ITS ANTI-DIABETIC POTENTIAL IN 3T3-L1 ADIPOCYTES

I am pleased to submit a revised manuscript entitled: "*Bioactive Properties of Fucoidan from Malaysian Brown Seaweed (Sargassum binderi) with an Assessment of Its Anti-Diabetic Potential in 3T3-L1 Adipocytes*", under consideration for publication in the *Journal of Food Science and Technology*.

2. We have performed the necessary revision based on the reviewer's comments and prepared a list of responses in a separate document. To facilitate the review process, all revised sections in the manuscript have been colored. Blue colors indicate changes made in response to Reviewer 1 meanwhile, red colors represent revisions addressing comments from Editor. We have carefully considered the reviewers' comments and tried our best to address every one of them.

3. All authors agree with the contents and revision of the revised manuscript and its resubmission to the journal. The authors would also like to state again that no part of the review has been published in any form elsewhere, and there is no conflict of interest involved.

We sincerely appreciate your consideration of our manuscript and look forward to your feedback. Thank you.

Yours sincerely,

....

Wan Aida Wan Mustapha
Corresponding author
Department of Food Science
Faculty of Science and Technology
National University of Malaysia (UKM)
43600 Selangor, Malaysia
